# *N*-(Thia­zol-2-yl)acetamide

**DOI:** 10.1107/S1600536808021442

**Published:** 2008-07-16

**Authors:** Uzma Yunus, Muhammad Kalim Tahir, Moazzam Hussain Bhatti, Wai-Yeung Wong

**Affiliations:** a Department of Chemistry, Allama Iqbal Open University, Islamabad, Pakistan; b Department of Chemistry, Hong Kong Baptist University, Waterloo Road, Kowloon Tong, Hong Kong

## Abstract

The title compound, C_5_H_6_N_2_OS, was synthesized from acetyl chloride and 2-amino­thia­zole in dry acetone. The asymmetric unit contains two mol­ecules. The crystal structure is stabilized by N—H⋯N and C—H⋯O hydrogen bonds.

## Related literature

For related literature, see: Raman *et al.* (2000 ▶); Wang *et al.* (2008 ▶); Yunus *et al.* (2007 ▶ 2008 ▶).
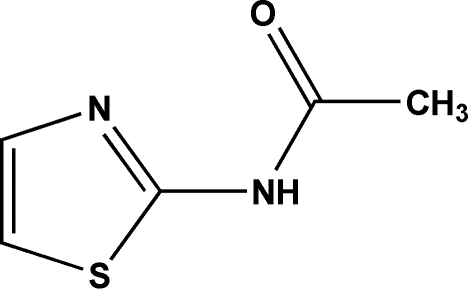


## Experimental

### 

#### Crystal data

C_5_H_6_N_2_OS
*M* *_r_* = 142.18
Monoclinic, 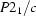

*a* = 16.0650 (12) Å
*b* = 11.3337 (8) Å
*c* = 7.0670 (5) Å
β = 101.908 (10)°
*V* = 1259.04 (16) Å^3^
*Z* = 8
Mo *K*α radiation
μ = 0.42 mm^−1^
*T* = 173 (2) K
0.30 × 0.26 × 0.22 mm

#### Data collection

Bruker SMART1000 CCD diffractometer
Absorption correction: multi-scan (*SADABS*; Bruker, 1999 ▶) *T*_min_ = 0.830, *T*_max_ = 1.000 (expected range = 0.757–0.911)
7429 measured reflections
3024 independent reflections
2602 reflections with *I* > 2σ(*I*)
*R*_int_ = 0.024

#### Refinement

*R*[*F* ^2^ > 2σ(*F* ^2^)] = 0.036
*wR*(*F* ^2^) = 0.104
*S* = 1.05
3024 reflections
163 parameters
H-atom parameters constrained
Δρ_max_ = 0.44 e Å^−3^
Δρ_min_ = −0.34 e Å^−3^

### 

Data collection: *SMART* (Bruker, 1999 ▶); cell refinement: *SAINT* (Bruker, 1999 ▶); data reduction: *SAINT*; program(s) used to solve structure: *SHELXS97* (Sheldrick, 2008 ▶); program(s) used to refine structure: *SHELXL97* (Sheldrick, 2008 ▶); molecular graphics: *Mercury* (Macrae *et al.*, 2006 ▶); software used to prepare material for publication: *SHELXTL* (Sheldrick, 2008 ▶).

## Supplementary Material

Crystal structure: contains datablocks I, global. DOI: 10.1107/S1600536808021442/wk2088sup1.cif

Structure factors: contains datablocks I. DOI: 10.1107/S1600536808021442/wk2088Isup2.hkl

Additional supplementary materials:  crystallographic information; 3D view; checkCIF report

**Fig. 1.**
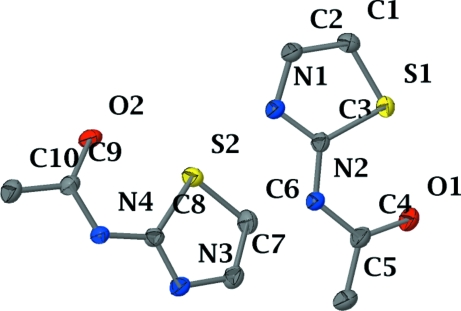
The molecular structure of (I) with ellipsoids drawn at the 50% probability level.

Enhanced figure: interactive version of Fig. 1

## Figures and Tables

**Table 1:** Hydrogen-bond geometry (Å, °)

*D*—H⋯*A*	*D*—H	H⋯*A*	*D*⋯*A*	*D*—H⋯*A*
N2—H2*B*⋯N3^i^	0.88	2.04	2.897 (2)	163
N4—H4*A*⋯N1^ii^	0.88	2.07	2.938 (2)	171
C2—H2*A*⋯O2^iii^	0.95	2.41	3.350 (2)	171
C7—H7*A*⋯O1^iv^	0.95	2.46	3.382 (2)	165

Symmetry codes: (i) 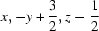; (ii) 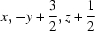; (iii) 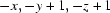; (iv) 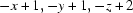.

**Fig. 2.**
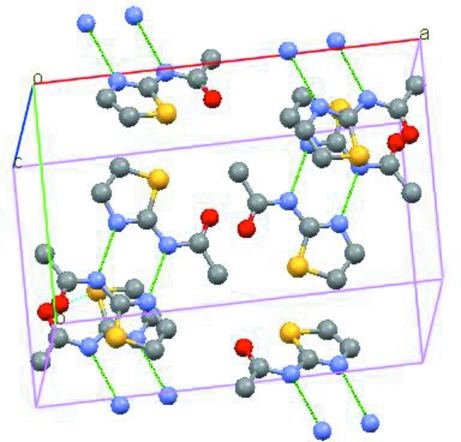
A packing diagram for (I) showing N—H···N hydrogen bonding.

## supplementary crystallographic information

### Comment

The thiazole ring and its derivatives are of great importance in biological systems due to their vast range of biological activities such as anti-inflammatory, analgesic and antipyretic (Raman *et al.*, 2000). On the other hand amide compounds have extensive applications in the pharmaceutical industry (Wang *et al.*, 2008). As a part of our research the title compound (I) has been synthesized and its crystal structure is reported herein (Yunus *et al.*, 2007; 2008).

The title compound (I) crystallizes in a monoclinic space group with two molecules in asymmetric unit. All the bond lengths and angles are within the normal ranges. The molecules are stabilized by intermolecular hydrogen bonds N—H···N, and C—H···O (Table 1, Fig 2).

### Experimental

A mixture of acetyl chloride (26 mmol) and 2-aminothiazole (26 mmol) was refluxed in dry acetone (60 ml) for two hours. After cooling, the mixture was poured into acidified cold water. The resulting yellow solid was filtered and washed with cold acetone. Single crystals of the title compound suitable for single-crystal *x*-ray analysis were obtained by recrystallization of the yellow solid from ethyl acetate.

### Crystal data

**Table:**

C_5_H_6_N_2_OS	*F*_000_ = 592
*M**_r_* = 142.18	*D*_x_ = 1.500 Mg m^−^^3^
Monoclinic, *P*2_1_/*c*	Mo *K*α radiation λ = 0.71073 Å
Hall symbol: -P 2ybc	Cell parameters from 7429 reflections
*a* = 16.0650 (12) Å	θ = 2.6–28.3º
*b* = 11.3337 (8) Å	µ = 0.42 mm^−^^1^
*c* = 7.0670 (5) Å	*T* = 173 (2) K
β = 101.908 (10)º	Block, pale yellow
*V* = 1259.04 (16) Å^3^	0.30 × 0.26 × 0.22 mm
*Z* = 8	

### Data collection

**Table:**

Bruker SMART1000 CCD diffractometer	3024 independent reflections
Radiation source: fine-focus sealed tube	2602 reflections with *I* > 2σ(*I*)
Monochromator: graphite	*R*_int_ = 0.024
*T* = 173(2) K	θ_max_ = 28.3º
ω and φ scans	θ_min_ = 2.6º
Absorption correction: multi-scan(SADABS; Bruker, 1999)	*h* = −21→18
*T*_min_ = 0.830, *T*_max_ = 1.000	*k* = −15→12
7429 measured reflections	*l* = −9→9

### Refinement

**Table:**

Refinement on *F*^2^	Secondary atom site location: difference Fourier map
Least-squares matrix: full	Hydrogen site location: inferred from neighbouring sites
*R*[*F*^2^ > 2σ(*F*^2^)] = 0.036	H-atom parameters constrained
*wR*(*F*^2^) = 0.104	*w* = 1/[σ^2^(*F*_o_^2^) + (0.0544*P*)^2^ + 0.5928*P*] where *P* = (*F*_o_^2^ + 2*F*_c_^2^)/3
*S* = 1.05	(Δ/σ)_max_ < 0.001
3024 reflections	Δρ_max_ = 0.44 e Å^−^^3^
163 parameters	Δρ_min_ = −0.34 e Å^−^^3^
Primary atom site location: structure-invariant direct methods	Extinction correction: none

### Special details

**Table:**

Geometry. All e.s.d.'s (except the e.s.d. in the dihedral angle between two l.s. planes) are estimated using the full covariance matrix. The cell e.s.d.'s are taken into account individually in the estimation of e.s.d.'s in distances, angles and torsion angles; correlations between e.s.d.'s in cell parameters are only used when they are defined by crystal symmetry. An approximate (isotropic) treatment of cell e.s.d.'s is used for estimating e.s.d.'s involving l.s. planes.
Refinement. Refinement of *F*^2^ against ALL reflections. The weighted *R*-factor *wR* and goodness of fit *S* are based on *F*^2^, conventional *R*-factors *R* are based on *F*, with *F* set to zero for negative *F*^2^. The threshold expression of *F*^2^ > σ(*F*^2^) is used only for calculating *R*-factors(gt) *etc*. and is not relevant to the choice of reflections for refinement. *R*-factors based on *F*^2^ are statistically about twice as large as those based on *F*, and *R*- factors based on ALL data will be even larger.

### Fractional atomic coordinates and isotropic or equivalent isotropic displacement parameters (Å^2^)

**Table:**

	*x*	*y*	*z*	*U*_iso_*/*U*_eq_	
C1	0.22544 (11)	0.26865 (16)	0.3481 (3)	0.0287 (4)	
H1A	0.2073	0.1965	0.2843	0.034*	
C2	0.17369 (11)	0.35988 (15)	0.3648 (3)	0.0276 (4)	
H2A	0.1143	0.3575	0.3127	0.033*	
C3	0.29472 (10)	0.43764 (14)	0.5196 (2)	0.0223 (3)	
C4	0.43344 (10)	0.50546 (16)	0.6861 (3)	0.0281 (4)	
C5	0.48077 (11)	0.61150 (18)	0.7800 (3)	0.0362 (4)	
H5A	0.5407	0.5910	0.8272	0.054*	
H5B	0.4560	0.6372	0.8887	0.054*	
H5C	0.4765	0.6755	0.6852	0.054*	
C6	0.28449 (12)	0.53644 (16)	1.0659 (3)	0.0313 (4)	
H6A	0.3085	0.4598	1.0867	0.038*	
C7	0.32581 (11)	0.63693 (16)	1.1264 (3)	0.0290 (4)	
H7A	0.3831	0.6374	1.1960	0.035*	
C8	0.20282 (10)	0.71411 (14)	0.9848 (2)	0.0227 (3)	
C9	0.06151 (10)	0.78011 (16)	0.8300 (3)	0.0277 (4)	
C10	0.00676 (12)	0.88668 (17)	0.7755 (3)	0.0381 (4)	
H10A	−0.0506	0.8618	0.7116	0.057*	
H10B	0.0312	0.9361	0.6872	0.057*	
H10C	0.0038	0.9318	0.8922	0.057*	
N1	0.21293 (9)	0.45737 (13)	0.4630 (2)	0.0254 (3)	
N2	0.34869 (8)	0.52329 (13)	0.6143 (2)	0.0252 (3)	
H2B	0.3273	0.5934	0.6293	0.030*	
N3	0.27956 (8)	0.73976 (13)	1.0806 (2)	0.0255 (3)	
N4	0.14391 (8)	0.80175 (12)	0.9218 (2)	0.0246 (3)	
H4A	0.1602	0.8756	0.9418	0.030*	
O1	0.46679 (8)	0.40975 (12)	0.6735 (2)	0.0386 (3)	
O2	0.03563 (8)	0.67920 (12)	0.7966 (2)	0.0380 (3)	
S1	0.32900 (3)	0.30062 (4)	0.45821 (7)	0.02647 (13)	
S2	0.18167 (3)	0.56560 (4)	0.94536 (7)	0.02913 (13)	

### Atomic displacement parameters (Å^2^)

**Table:**

	*U*^11^	*U*^22^	*U*^33^	*U*^12^	*U*^13^	*U*^23^
C1	0.0280 (8)	0.0239 (8)	0.0334 (9)	−0.0035 (6)	0.0047 (7)	−0.0030 (7)
C2	0.0213 (8)	0.0266 (8)	0.0329 (9)	−0.0019 (6)	0.0008 (6)	−0.0002 (7)
C3	0.0200 (7)	0.0211 (7)	0.0250 (8)	0.0019 (6)	0.0030 (6)	0.0021 (6)
C4	0.0200 (8)	0.0303 (9)	0.0322 (9)	0.0014 (6)	0.0016 (7)	0.0026 (7)
C5	0.0232 (8)	0.0368 (10)	0.0448 (11)	−0.0038 (7)	−0.0015 (7)	−0.0044 (8)
C6	0.0300 (9)	0.0250 (8)	0.0376 (10)	0.0057 (7)	0.0038 (7)	0.0037 (7)
C7	0.0237 (8)	0.0296 (9)	0.0321 (9)	0.0033 (7)	0.0024 (7)	0.0052 (7)
C8	0.0217 (7)	0.0215 (7)	0.0247 (8)	−0.0020 (6)	0.0044 (6)	0.0014 (6)
C9	0.0184 (7)	0.0280 (9)	0.0352 (9)	−0.0010 (6)	0.0021 (6)	0.0005 (7)
C10	0.0240 (8)	0.0327 (10)	0.0540 (12)	0.0040 (7)	−0.0004 (8)	0.0011 (9)
N1	0.0185 (6)	0.0249 (7)	0.0314 (8)	0.0000 (5)	0.0016 (5)	−0.0005 (6)
N2	0.0184 (6)	0.0222 (7)	0.0329 (8)	0.0005 (5)	0.0005 (5)	−0.0019 (6)
N3	0.0200 (6)	0.0242 (7)	0.0305 (8)	−0.0007 (5)	0.0009 (5)	0.0032 (6)
N4	0.0182 (6)	0.0195 (7)	0.0342 (8)	−0.0011 (5)	0.0008 (5)	−0.0003 (5)
O1	0.0232 (6)	0.0317 (7)	0.0557 (9)	0.0058 (5)	−0.0039 (6)	−0.0007 (6)
O2	0.0220 (6)	0.0276 (7)	0.0590 (9)	−0.0047 (5)	−0.0042 (6)	−0.0023 (6)
S1	0.0229 (2)	0.0210 (2)	0.0349 (2)	0.00316 (14)	0.00479 (16)	−0.00010 (15)
S2	0.0261 (2)	0.0205 (2)	0.0385 (3)	−0.00185 (15)	0.00132 (17)	−0.00081 (16)

### Geometric parameters (Å, °)

**Table:**

C1—C2	1.347 (2)	C6—S2	1.7271 (19)
C1—S1	1.7236 (18)	C6—H6A	0.9500
C1—H1A	0.9500	C7—N3	1.384 (2)
C2—N1	1.385 (2)	C7—H7A	0.9500
C2—H2A	0.9500	C8—N3	1.311 (2)
C3—N1	1.311 (2)	C8—N4	1.381 (2)
C3—N2	1.379 (2)	C8—S2	1.7284 (17)
C3—S1	1.7326 (16)	C9—O2	1.223 (2)
C4—O1	1.221 (2)	C9—N4	1.371 (2)
C4—N2	1.366 (2)	C9—C10	1.497 (2)
C4—C5	1.502 (3)	C10—H10A	0.9800
C5—H5A	0.9800	C10—H10B	0.9800
C5—H5B	0.9800	C10—H10C	0.9800
C5—H5C	0.9800	N2—H2B	0.8800
C6—C7	1.343 (3)	N4—H4A	0.8800
			
C2—C1—S1	110.78 (13)	N3—C7—H7A	122.2
C2—C1—H1A	124.6	N3—C8—N4	121.06 (15)
S1—C1—H1A	124.6	N3—C8—S2	115.59 (12)
C1—C2—N1	115.51 (15)	N4—C8—S2	123.35 (12)
C1—C2—H2A	122.2	O2—C9—N4	121.01 (16)
N1—C2—H2A	122.2	O2—C9—C10	123.13 (16)
N1—C3—N2	121.20 (15)	N4—C9—C10	115.86 (15)
N1—C3—S1	115.26 (12)	C9—C10—H10A	109.5
N2—C3—S1	123.49 (12)	C9—C10—H10B	109.5
O1—C4—N2	121.52 (16)	H10A—C10—H10B	109.5
O1—C4—C5	123.60 (15)	C9—C10—H10C	109.5
N2—C4—C5	114.88 (15)	H10A—C10—H10C	109.5
C4—C5—H5A	109.5	H10B—C10—H10C	109.5
C4—C5—H5B	109.5	C3—N1—C2	109.91 (14)
H5A—C5—H5B	109.5	C4—N2—C3	123.68 (15)
C4—C5—H5C	109.5	C4—N2—H2B	118.2
H5A—C5—H5C	109.5	C3—N2—H2B	118.2
H5B—C5—H5C	109.5	C8—N3—C7	109.65 (15)
C7—C6—S2	110.75 (13)	C9—N4—C8	123.68 (14)
C7—C6—H6A	124.6	C9—N4—H4A	118.2
S2—C6—H6A	124.6	C8—N4—H4A	118.2
C6—C7—N3	115.69 (15)	C1—S1—C3	88.54 (8)
C6—C7—H7A	122.2	C6—S2—C8	88.31 (8)

### Hydrogen-bond geometry (Å, °)

**Table:**

*D*—H···*A*	*D*—H	H···*A*	*D*···*A*	*D*—H···*A*
N2—H2B···N3^i^	0.88	2.04	2.897 (2)	163
N4—H4A···N1^ii^	0.88	2.07	2.938 (2)	171
C2—H2A···O2^iii^	0.95	2.41	3.350 (2)	171
C7—H7A···O1^iv^	0.95	2.46	3.382 (2)	165

Symmetry codes: (i) *x*, −*y*+3/2, *z*−1/2; (ii) *x*, −*y*+3/2, *z*+1/2; (iii) −*x*, −*y*+1, −*z*+1; (iv) −*x*+1, −*y*+1, −*z*+2.

## References

[bb1] Bruker (1999). *SMART*, *SAINT* and *SADABS* Bruker AXS Inc., Madison, Wisconsin, USA.

[bb2] Macrae, C. F., Edgington, P. R., McCabe, P., Pidcock, E., Shields, G. P., Taylor, R., Towler, M. & van de Streek, J. (2006). *J. Appl. Cryst.* **39**, 453–457.

[bb3] Raman, R., Razavi, H. & Kelly, J. W. (2000). *Org. Lett.* **2**, 3289–3292. 10.1021/ol000178q 11029192

[bb4] Sheldrick, G. M. (2008). *Acta Cryst.* A**64**, 112–122. 10.1107/S0108767307043930 18156677

[bb5] Wang, X.-J., Yang, Q., Liu, F. & You, Q.-D. (2008). *Synth. Commun.* **38**, 1028–1035.

[bb6] Yunus, U., Tahir, M. K., Bhatti, M. H., Ali, S. & Helliwell, M. (2007). *Acta Cryst.* E**63**, o3690.

[bb7] Yunus, U., Tahir, M. K., Bhatti, M. H. & Wong, W.-Y. (2008). *Acta Cryst.* E**64**, o722. 10.1107/S1600536808006752 PMC2961019 21202112

